# Identification of early Alzheimer’s disease subclass and signature genes based on PANoptosis genes

**DOI:** 10.3389/fimmu.2024.1462003

**Published:** 2024-11-22

**Authors:** Wenxu Wang, Jincheng Lu, Ningyun Pan, Huiying Zhang, Jingcen Dai, Jie Li, Cheng Chi, Liumei Zhang, Liang Wang, Mengying Zhang

**Affiliations:** ^1^ School of Medical Information and Engineering, Xuzhou Medical University, Xuzhou, Jiangsu, China; ^2^ College of Life Science, Xuzhou Medical University, Xuzhou, Jiangsu, China; ^3^ School of Mathematics and System Science, Shandong University of Science and Technology, Qingdao, Shandong, China; ^4^ Laboratory Medicine, Guangdong Provincial People’s Hospital (Guangdong Academy of Medical Sciences), Southern Medical University, Guangzhou, Guangdong, China

**Keywords:** Alzheimer’s disease, PANoptosis, molecular subtypes, drug gene interactions, therapeutic targets of early AD

## Abstract

**Introduction:**

Alzheimer’s disease (AD) is one of the most prevalent forms of dementia globally and remains an incurable condition that often leads to death. PANoptosis represents an emerging paradigm in programmed cell death, integrating three critical processes: pyroptosis, apoptosis, and necroptosis. Studies have shown that apoptosis, necroptosis, and pyroptosis play important roles in AD development. Therefore, targeting PANoptosis genes might lead to novel therapeutic targets and clinically relevant therapeutic approaches. This study aims to identify different molecular subtypes of AD and potential drugs for treating AD based on PANoptosis.

**Methods:**

Differentially expressed PANoptosis genes associated with AD were identified via Gene Expression Omnibus (GEO) dataset GSE48350, GSE5281, and GSE122063. Least Absolute Shrinkage and Selection Operator (LASSO) regression was employed to construct a risk model linked to these PANoptosis genes. Consensus clustering analysis was conducted to define AD subtypes based on these genes. We further performed gene set variation analysis (GSVA), functional enrichment analysis, and immune cell infiltration analysis to investigate differences between the identified AD subtypes. Additionally, a protein-protein interaction (PPI) network was established to identify hub genes, and the DGIdb database was consulted to identify potential therapeutic compounds targeting these hub genes. Single-cell RNA sequencing analysis was utilized to assess differences in gene expression at the cellular level across subtypes.

**Results:**

A total of 24 differentially expressed PANoptosis genes (APANRGs) were identified in AD, leading to the classification of two distinct AD subgroups. The results indicate that these subgroups exhibit varying disease progression states, with the early subtype primarily linked to dysfunctional synaptic signaling. Furthermore, we identified hub genes from the differentially expressed genes (DEGs) between the two clusters and predicted 38 candidate drugs and compounds for early AD treatment based on these hub genes. Single-cell RNA sequencing analysis revealed that key genes associated with the early subtype are predominantly expressed in neuronal cells, while the differential genes for the metabolic subtype are primarily found in endothelial cells and astrocytes.

**Conclusion:**

In summary, we identified two subtypes, including the AD early synaptic abnormality subtype as well as the immune-metabolic subtype. Additionally, ten hub genes, SLC17A7, SNAP25, GAD1, SLC17A6, SLC32A1, PVALB, SYP, GRIN2A, SLC12A5, and SYN2, were identified as marker genes for the early subtype. These findings may provide valuable insights for the early diagnosis of AD and contribute to the development of innovative therapeutic strategies.

## 1 Introduction

Alzheimer’s disease(AD) is the main cause of dementia and is quickly becoming one of the most lethal, expensive, and burdening diseases of this century (1), and its prevalence of dementia will triple worldwide by 2050 (2). The clinical features of AD are characterized by progressive loss of memory, learning, language, and cognitive functions (3), and the primary pathological characteristics of AD are the buildup of amyloid-β (Aβ) plaque and intraneuronal neurofibrillary tangle (NFT) (4). Aβ plaques form owing to the successive enzymatic breakdown of amyloid precursor protein by β-secretase and γ-secretase. In recent years, important advances have been made in understanding the underlying pathology, identifying multiple disease-causing and protective genes, recognizing new blood and imaging biomarkers, and disease-improving treatments (5–8), the underlying mechanisms of AD remain incompletely understood, and current therapeutic options remain inadequate (9). Consequently, early diagnosis and intervention are essential for improving patient outcomes, and identifying molecular subtypes could help uncover targeted therapies for AD.

PANoptosis, an emerging concept in programmed cell death (PCD), involves the interaction and regulation of three processes, apoptosis, necroptosis, and pyroptosis, and reliance on any one of these processes alone cannot fully explain the phenomenon (10–12). While these processes individually play crucial roles in AD pathology, their combined role under the umbrella of PANoptosis remains unclear. Research shows that apoptosis and necroptosis are highly prevalent in the neurons and glial cells of AD patients, contributing to disease progression (13). Pyroptosis, the inflammatory variant of programmed cell death, acts as a catalyst for neuronal death in AD by activating NLRP3 and caspases, leading to the secretion of IL-1 and IL-18 (14, 15). Despite the importance of these individual pathways in AD, how PANoptosis as a whole influences AD progression is still poorly understood.

**Figure 1:**
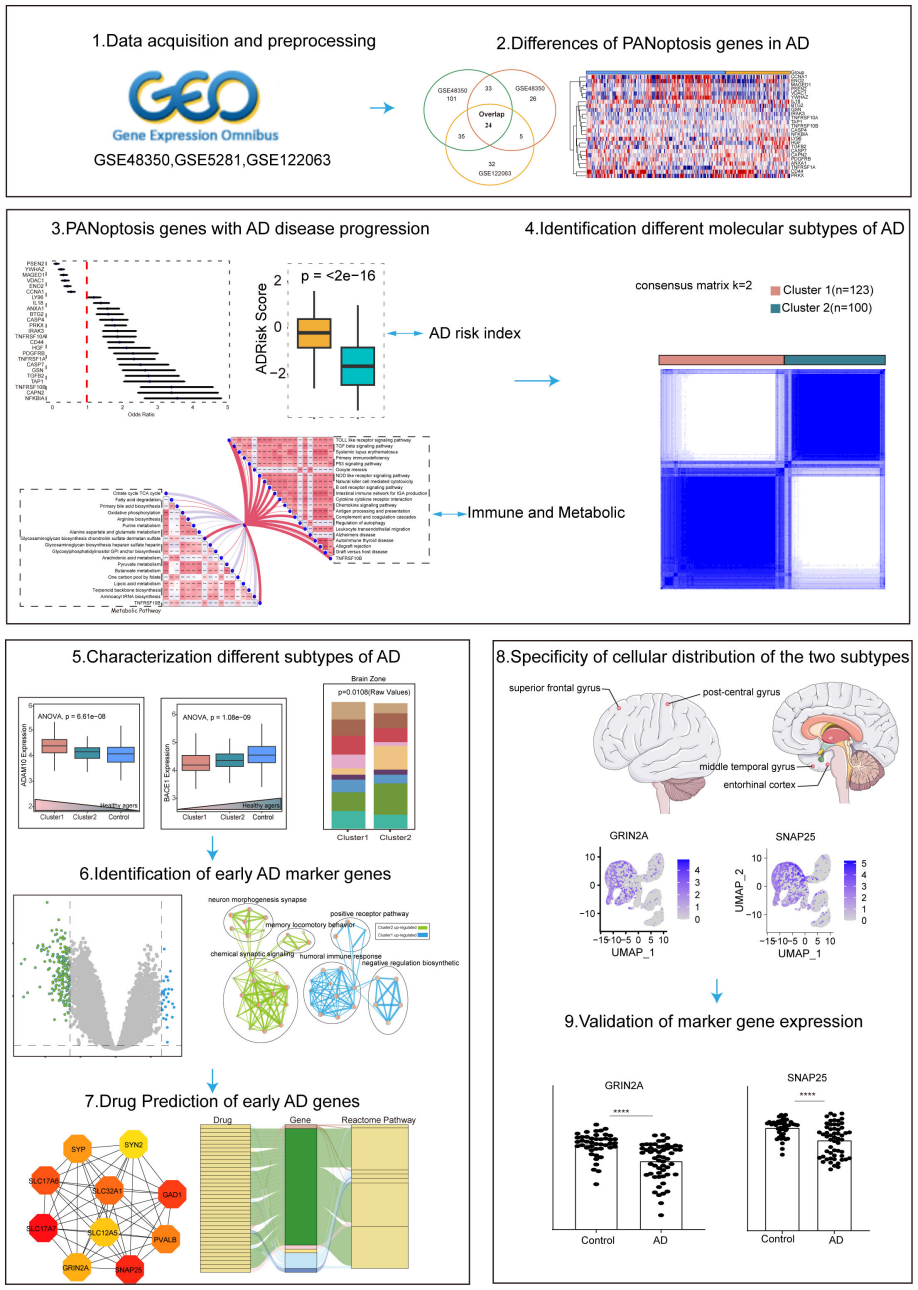
Flowchart of the research.

Recent advancements in gene expression profiling, particularly through single-cell sequencing, have provided new insights into the complexity of AD (16, 17). By analyzing the expression of PANoptosis-related genes (APANRGs) in AD and normal tissues, we identified key genes associated with AD progression. Our study classified AD into two subtypes based on these genes, each subtype exhibiting distinct disease progression patterns. The early subtype is primarily linked to disrupted synaptic signaling, while the other subtype, associated with metabolic dysfunction, involves differentially expressed genes in endothelial cells and astrocytes. We also identified hub genes between these clusters and predicted candidate drugs through the Drug Gene Interaction Database. Single-cell analysis revealed that key genes in the early subtype were predominantly expressed in neurons, whereas the metabolic subtype was associated with endothelial and astrocyte expression. The workflow is illustrated in **Figure 1**. In summary, our findings provide potential new avenues for the early diagnosis and treatment of AD.

## 2 Materials and methods

### 2.1 Acquisition and preprocessing of AD datasets

Gene expression data of AD patients were obtained from the GEO database (http://www.ncbi.nlm.nih.gov/geo). We selected three datasets (GSE48350, GSE122063, and GSE5281, postmortem brain tissue transcriptome analysis of AD patients) for the next analysis. We mainly focus on healthy individuals and AD patients, excluding the other disease individuals. The three datasets cover different brain regions and disease process stages. Additional single-cell data from different brain regions of AD were obtained to check the hub gene expression status of GEO, including GSE147528, GSE188545, and GSE160936. The detailed information on selected datasets is shown in **Tables 1**, **2** and **Supplementary Table S1**. The sample of AD patients contained multiple brain regions such as the entorhinal cortex, hippocampus, medial temporal gyrus, posterior cingulate, superior frontal gyrus, and primary visual cortex. All control human AD samples were obtained from the normal-aged brain.

**Table 1:** Difference expression datasets referenced in this study.

GSE dataset	Organism	Sample number	PMID	Platform	Type
GSE48350	Homo sapiens	Control:173 AD:80	PMID: 18832152	GPL570	Bulk-seq
GSE122063	Homo sapiens	Control:44 AD:56	PMID: 30990880	GPL16699	Bulk-seq
GSE5281	Homo sapiens	Control:74 AD:87	PMID: 17077275	GPL570	Bulk-seq

**Table 2:** Single-cell sequencing datasets from GEO.

GSE dataset	Organism	Sample number	PMID	Platform	Type
GSE147528	Homo sapiens	EC:10 SFG:10	PMID: 33432193	GPL24676	scRNA-seq
GSE188545	Homo sapiens	MTG:6	PMID: 36865305	GPL24676	scRNA-seq
GSE160936	Homo sapiens	SSC:6	PMID: 34767070	GPL20301	scRNA-seq

### 2.2 Identification of differentially expressed PANoptosis−related genes

The 509 PANoptosis genes were obtained from the literature (18, 19), including apoptosis, pyroptosis, and necroptosis, and they are presented in **Supplementary Table S2** and **Supplementary Figures S1A, B**. GSE48350, GSE122063, and GSE5281 datasets were downloaded via R package “GEOquery”. Differentially expressed genes in the GSE48350, GSE122063, and GSE5281 datasets were identified using the “limma” (20) R package with |Log2FC| > 0.1375, |Log2FC| > 0.5, and |Log2FC| > 0.5, respectively. Meanwhile, all three datasets satisfied FDR<0.05. Subsequently, PANoptosis-related DEG expression was extracted from three differentially expressed gene datasets.

### 2.3 PANoptosis-related genes correlation analysis

The correlation of 24 PANoptosis-related DEG’s expression in all samples and AD samples was calculated. The correlations were performed by Spearman’s correlation analysis and visualized by the “corrplot” R package (21). Twenty confirmed AD-related genes were downloaded from the ADSP database (https://adsp.niagads.org/gvc-top-hits-list/). Correlation calculations were performed between these and 24 PANoptosis-related DEGs.

### 2.4 Risk prediction in AD patients based on PANoptosis-related DEGs

Univariate logistic regression analysis was used to identify PANoptosis-related DEGs significantly associated with AD. LASSO regression models were applied using the R package glmnet version 4.1.8 to reduce dimensionality and select significant genes. First, models for LASSO were constructed with PANoptosis-related DEG expression data and binary discrete variables (AD and normal). Next, the model was cross-validated to obtain the best lambda value with a regression penalty score of -1.2927, and the genes screened by LASSO were extracted for multivariate logistic regression analysis. Finally, the AD risk score for each sample was calculated based on the prediction function. We generated the AdRisk score via regression coefficients according to the following formula:


$$\text{ADRisk score}=\sum_{i}^{n}gene_{i}*coef_{i}$$

where *gene_i_* represents the PANoptosis-related DEG expression and *coef_i_* represents the LASSO coefficient of genes, respectively.

### 2.5 Gene set variation analysis

Gene Set Variation Analysis (GSVA) (22) analysis was applied to explore the difference in biological pathways between distinct patterns according to the enrichment score. GSVA version 1.48.3 was applied to perform functional enrichment analysis to obtain the enrichment pathways score, the parameters are set as follows: mx.diff=FALSE, verbose=FALSE, parallel.sz =1,method=“ssgsea”. We downloaded “c2.cp. kegg.v7.4.symbols.gmt” from the MsigDB (23) database for analysis, including immune pathways and metabolic pathways. Additional PANoptosis-related DEGs were analyzed for correlation with pathway scores, with p< 0.05 defined as significant gene-pathway correlation.

### 2.6 Classification and analysis of AD subtypes

#### 2.6.1 Consistent clustering of AD patients

To explore whether PANoptosis-related DEGs can be used for subtypes of AD samples, we next used the ConsensusClusterPlus package (24) to identify the optimum number of clusters in AD patients. The expression profiles were firstly normalized by subtracting the median and then subjected to this R package. We used hierarchical clustering with Pearson correlation as the similarity metric, with k values ranging from 1 to 6. We selected the best solution for the consensus matrix by considering the relative change in area under the CDF curve.

#### 2.6.2 Immune cell infiltration in AD subtypes

The CIBERSORT (25) algorithm is a machine learning method based on linear support vector regression for evaluating the proportion of 22 immune cells in AD patient samples. With the parameter setting perm = 100, this experiment simulates the transcriptional signature matrix of 22 immune cells such as T cells, B cells, monocytes, macrophages, mast cells, dendritic cells, and neutrophils.

#### 2.6.3 Characterizing the functional heterogeneity of AD subtypes

Next, the differentially expressed genes of the 2 clusters were identified using the Limma package using strict thresholds: adjusted p<0.05 and |log2FC|>1.3. The R package clusterProfiler was used to conduct GO biological process analysis [32]. Go terms with p-value< 0.05 were considered as significant enrichment. KEGG pathway analyses of DEGs in the two clusters were obtained in the *DAVID* database, and p<0.05 was considered significant enrichment. Cytoscape (26) plugins EnrichmentMap (27) and AutoAnnotate (28) were used to visualize similar BP terms with stringent similarity scores.

#### 2.6.4 PPI network construction in subtypes

PPI networks were constructed by importing genes into the search tool for retrieving gene interactions (STRING, www.string-db.org) (29). Cytoscape (version 3.7.2) was used to visualize the network, while the cytoHubba plugin (30)was used to sort the genes in the network according to their topological properties. The algorithm selected in cytoHubba was MCC. Hub genes were those with the top 10 MCC values.

#### 2.6.5 Drug prediction based on subtyped highly expressed genes

The DGIdb database (31) (Drug-Gene Interaction database, http://dgidb.org/), is a drug-gene interaction database that provides information on the association of genes with their known or potential drugs, including Alzheimer’s disease-related genes (31). Selected hub genes, which were considered potential drug targets for the treatment of AD, were imported into DGIdb to explore existing drugs or compounds. Gene-drug interactions were shown as results using the R packages ggplot2 and riverplot.

### 2.7 Characterizing the landscape of different brain regions in AD at the single-cell level

#### 2.7.1 Analysis of scRNA-sequencing data of AD

R (version 4.3.1) and the Seurat R package (32) (version 4.3.0.1) were used for the analyses. For each sample for 4 brain regions, the gene and count features were identified, and cells with less than 200 or more than 4000 features were filtered. Then, cells with mitochondrial RNA percentage > 15 were further removed. Potential doublets identified were also removed from further analyses. Furthermore, the top 2000 highly variable genes in single cells were identified after controlling for the expression. Principal component analysis with variable genes was used to identify significant principal components (PCs) based on the jackStraw function. With a resolution of 0.3, cells were clustered using the ‘FindClusters’ function. Differentially expressed genes (DEGs) in each cluster were identified using the “FindAllMarkers” function. Subsequently, a few classical markers of brain cell subsets were obtained from a previous study (33) and CellMarker 2.0 (34), and manually labeled according to the expression of maker genes.

#### 2.7.2 Cell-cell communication analysis

We used CellChat (35) to explore the interactions between cell-to-cell communication, which comprises ligand-receptor interaction databases to assess intercellular communication networks based on scRNA-seq data from different cell clusters. netVisual_bubble was used to demonstrate multiple ligand-receptor-mediated cellular interactions (L-R pairs).

### 2.8 Statistical analysis

All statistical analyses were carried out in R 4.3.1 and GraphPad Prism 9. Statistical tests such as t-test, chi-square test, and ANOVA were used to test the differences between the 2 subgroup samples. Univariate and multivariate logistic regression analyses to assess the diagnostic value of AD risk models. All statistical tests were two-sided and p< 0.05 was defined as a significant difference.

## 3 Results

### 3.1 Defining the expression of PANoptosis genes in AD

**Figure 2:**
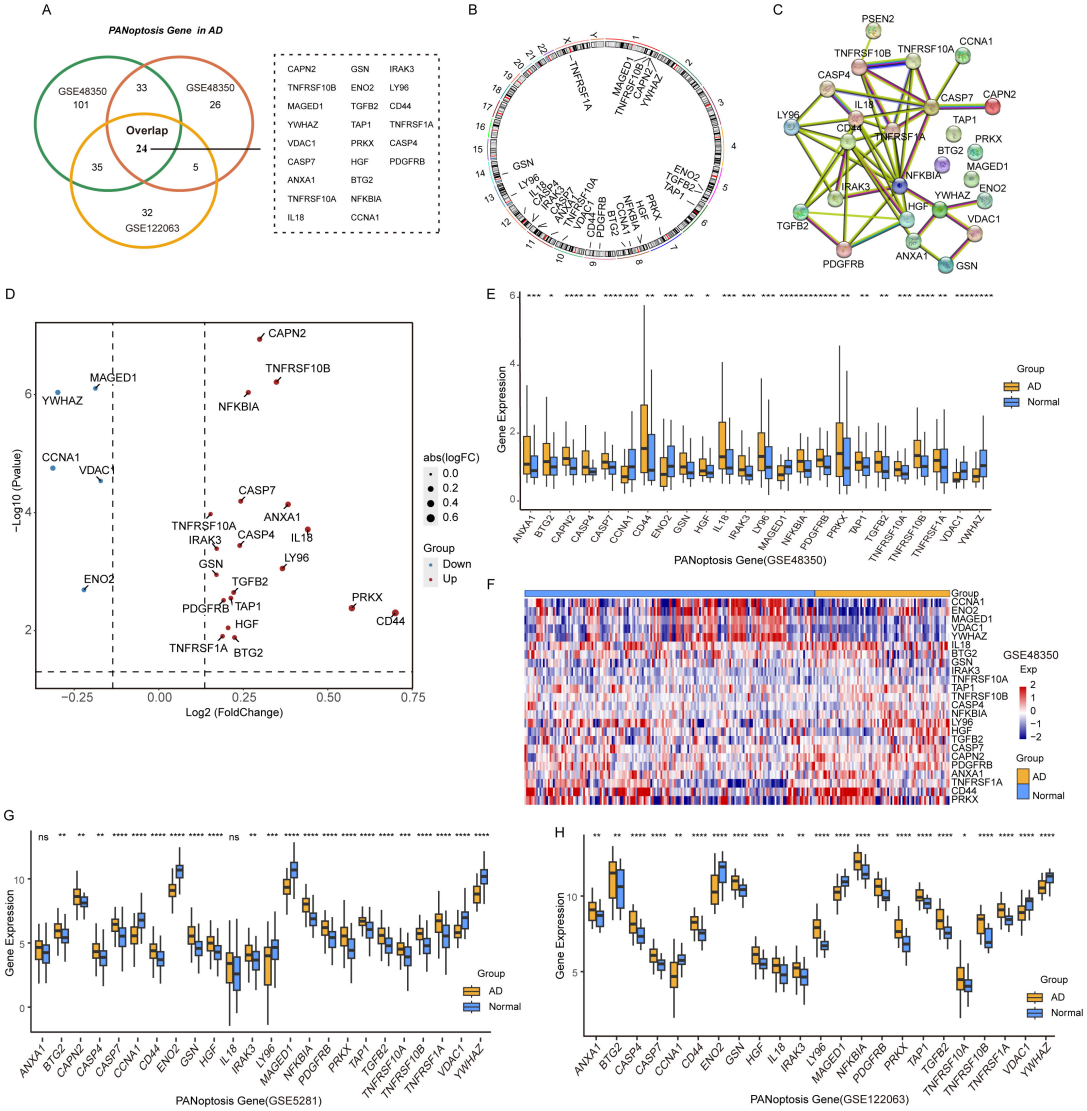
Characteristics and differences of PANoptosis genes in AD. **(A)** Venn plot of the differentially expressed PANoptosis genes in the three GEO datasets. **(B)** The location of 24 PANoptosis genes and PSEN2 on 23 chromosomes. **(C)** PPI network showing the interactions of the PANoptosis-related genes. **(D)** The volcano plot of the 24 differentially expressed PANoptosis-related genes in the GSE48350 dataset. Up-regulated, red; Down-regulated, blue. **(E)** A box plot of the 24 PANoptosis genes in the GSE48350 dataset. Normal, blue; AD, yellow. P values were shown as: *P< 0.05; **P< 0.01; ***P< 0.001; ****P< 0.0001. **(F)** A heatmap plot of the 24 PANoptosis genes. **(G)** A box plot of the 24 PANoptosis genes in the GSE5281 dataset. **(H)** A box plot of the 24 PANoptosis genes in the GSE122063 dataset.

The PANoptosis gene sets include 381 apoptosis-related genes, 27 pyroptosis-related genes, and 160 necroptosis-related genes (**Supplementary Figures S1A, B**). Differential expression analysis of PANoptosis genes in AD revealed 88 differentially expressed genes (DEGs) in the GSE48350 dataset, 193 DEGs in the GSE5281 dataset, and 96 DEGs in the GSE122063 dataset (**Table 3**). By comparing these gene sets, we identified 24 overlapping genes, which we defined as PANoptosis-related genes for AD (APANRGs) (**Figure 2A**). Chromosomal mapping of these 24 APANRGs showed they are predominantly located on autosomes (**Figure 2B**). To further explore the crosstalk of 24 APANRGs, we conducted a PPI network using the STRING database, and the result shows a tight interaction across the 24 genes (**Figure 2C**). The volcano plot shows the differential expression pattern of 24 APANRGs in the GSE48350 dataset (**Figures 2D–F**). Among the APANRGs, 5 genes (CCNA1, ENO2, MAGED1, VDAC1, YWHAZ) were down regulated while 19 genes (ANXA1, BTG2, CAPN2, CASP4, CASP7, CD44, GSN, HGF, IL18, IRAK3, LY96, NFKBIA, PDGFRB, PRKX, TAP1, TGFB2, TNFRSF10A, TNFRSF10B, TNFRSF1A) were up regulated in AD.

**Table 3:** PANoptosis genes identified in GSE datasets.

GSE dataset	DEGs	DEG_PANoptosis
GSE48350	3064	88
GSE122063	4920	96
GSE5281	7037	193

To validate these findings, we analyzed the expression patterns of the 24 APANRGs in the GSE5281 and GSE122063 datasets. Consistently, five genes were downregulated in both datasets, while LY96 showed an opposing expression pattern between the GSE48350 and GSE5281 datasets (**Figures 2G, H**). The remaining upregulated genes displayed consistent differential expression across all three datasets, suggesting that these 24 APANRGs exhibit stable differential expression in AD.

### 3.2 Association between APANRG expression and AD disease progression

**Figure 3:**
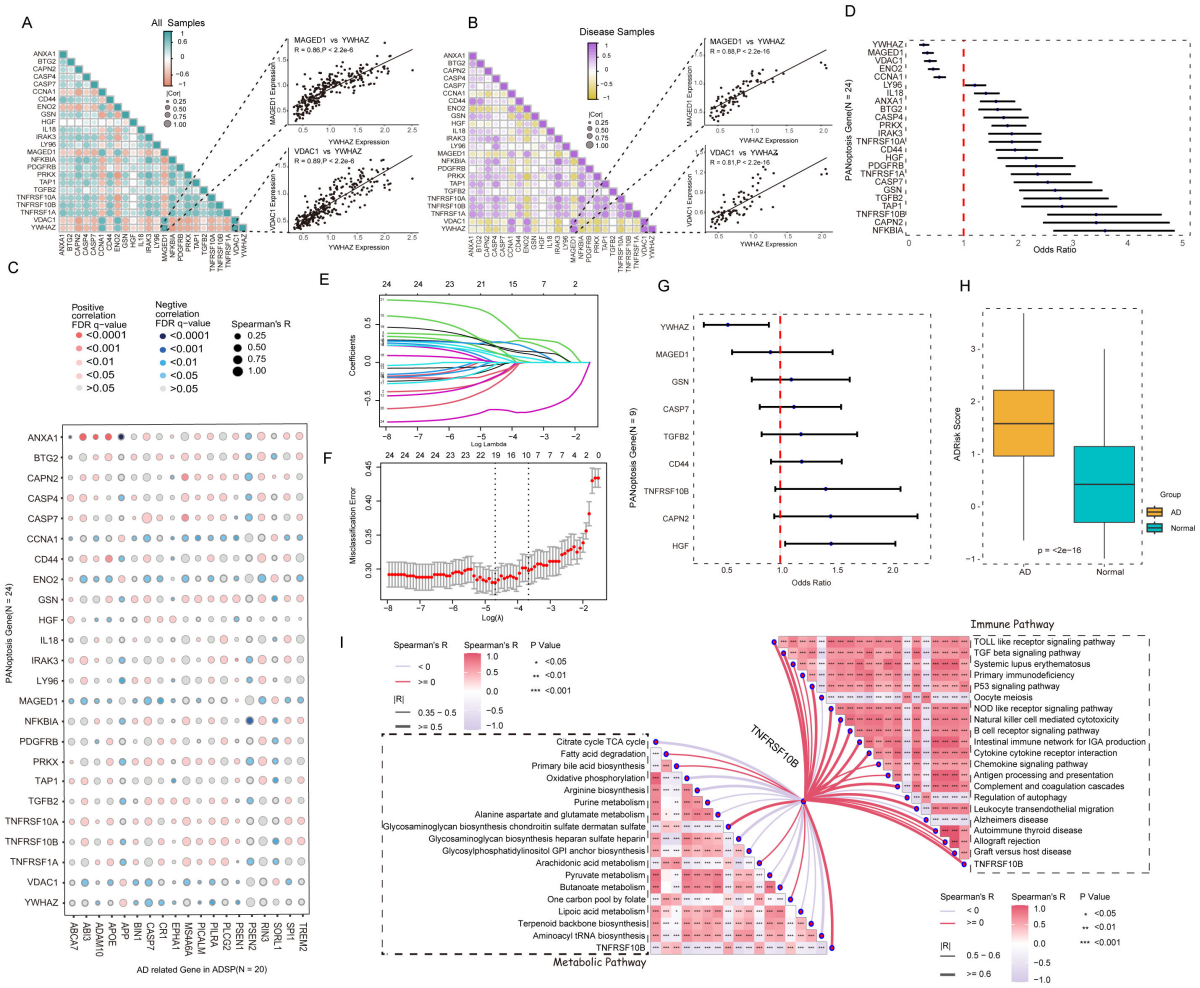
Association of APANRGs expression with AD disease progression. **(A)** Correlation analysis of APANRGs in GSE96804; All samples. **(B)** Correlation analysis of APANRGs in GSE96804; Disease samples. **(C)** Correlation analysis of AD susceptibility genes with APANRGs. **(D)** Forest plot for univariate logistic regression analysis of 24 APANRGs. **(E)** LASSO coefficient for 24 APANRGs genes. **(F)** Cross-validation curves for 24 APANRGs genes. **(G)** Forest plot of multivariate logistic regression analysis for 24 APANRGs. **(H)** Risk score of normal and AD group. **(I)** Enrichment analysis of TNFRSF10B gene in metabolic and immune pathways.

24 APANRGs expression correlation in all samples and AD samples were calculated via R package corrplot (21). We found that 24 APANRGs were associated with each other. For instance, YWHAZ was correlated with MAGED1 and VDAC1 in all cases and AD cases (**Figures 3A, B**, spearman R = 0.86,0.89 in all samples, respectively, and p< 2.2e-6, p< 2.2e-6, respectively. spearman R = 0.88,0.81 in AD samples, respectively, p< 2.2e-16, p< 2.2e-16, respectively). The Alzheimer’s Disease Sequencing Project (ADSP, https://adsp.niagads.org/) database contains 20 AD susceptibility genes (36). Correlation analyses demonstrated that the 24 APANRGs were strongly associated with the expression of these AD susceptibility genes (**Figure 3C**).

Univariate logistic regression analysis was applied to screen AD-related PANoptosis genes among the above 24 APANRGs. All 24 genes met the significance of p<0.05, down-regulated genes with OR< 1 were defined as AD-protective genes, and up-regulated genes with OR > 1 were significantly associated with AD progression (**Figure 3D**). Next, we applied all 24 APANRGs into LASSO regression and included 9 genes in the prediction model based on the optimal λ value (**Figures 3E, F**). Multivariate logistic regression analysis showed that HGF and YWHAZ were significantly associated with AD progression (**Figure 3G**). We then generated the AD risk score (AdRisk) via regression coefficients according to the following formula: AdRisk =-1.2927 + 0.2807 * CAPN2+ 0.0690 * CASP7 + 0.1229 * CD44 + 0.0215 * GSN+0.2170 * HGF - 0.0057 * MAGED1 + 0.1487 *TGFB2 + 0.3159 * TNFRSF10B - 0.6568 * YWHAZ.

The AD group had significantly higher AdRisk scores than the control group (p< 2e-16, **Figure 3H**), suggesting that APANRGs play a role in AD progression. Further analysis of TNFRSF10B, a key gene in the risk model, indicated that it is strongly associated with immune and metabolic pathways, as well as with the regulation of Alzheimer’s disease-related pathways (**Figure 3I**). YWHAZ and TNFRSF10A were also closely linked to immune and metabolic pathways (**Supplementary Figures S2A, B**). These findings suggest that APANRGs may contribute to AD progression through multiple biological pathways.

### 3.3 Identification and analysis of different molecular subtypes of AD based on PANoptosis genes

**Figure 4:**
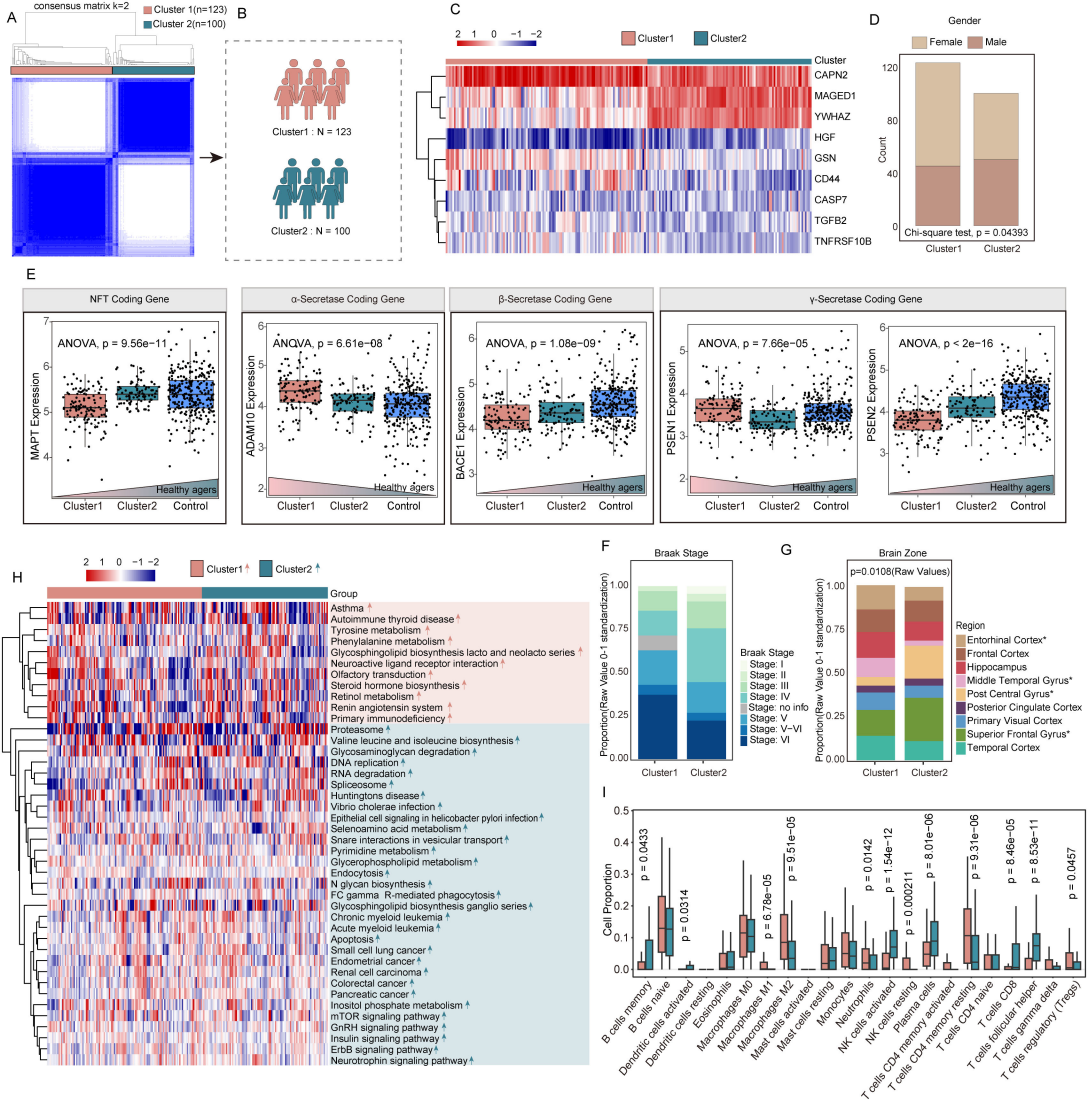
Identification and analysis of different molecular subtypes of AD based on PANoptosis genes. **(A, B)** 223 AD patients in three datasets were grouped into two clusters according to the consensus clustering matrix (k = 2). **(C)** Heatmap of the two clusters divided by the 14 APANRGs. **(D)** Proportion of sex between two subclasses. **(E)** Comparison of coding gene of NFT, gamma-secretase, beta-secretase, and alpha-secretase between each subclass. **(F)** Proportion of Braak stage between two subclasses. **(G)** Proportion of brain zone between two subclasses. **(H)** Heatmap of GSVA analysis showed representative KEGG pathways. **(I)** The immune cell infiltration score in two clusters.

Based on 24 APANRGs, consensus clustering classified the gene expression profiles for 223 AD samples (including GSE48350, GSE122063, and GSE5281) after removing the batch effect into distinct subclasses. The AD samples were categorized into two to six subclasses (**Supplementary Figure S3**). According to the CDF plot and consensus matrix heatmap, k=2 was determined as the optimal number of clusters. Finally, 123 cases were included in Cluster 1, and 100 cases were included in Cluster 2, respectively (**Figures 4A, B**). Moreover, we analyzed the inter-cluster expression of the 9 AdRisk genes and found that 8 of the 9 genes (CAPN2, CASP7, CD44, GSN, MAGED1, TGFB2, TNFRSF10B, YWHA) showed significant expression differences between clusters (**Figure 4C**, **Supplementary Figure S3**). There was a significant gender difference between the two subtypes, with a greater proportion of women than men overall (p = 0.04393, **Figure 4D**).

To explore the molecular differences between these clusters, we analyzed genes involved in alpha-secretase, gamma-secretase, and neurofibrillary tangle (NFT) formation. The NFT coding gene in Cluster 1 was notably lower compared with that in Cluster 2 and healthy agers (p = 9.56e-11; **Figure 4E**). Compared with Cluster 2, alpha-secretase coding gene-ADAM10, gamma-secretase coding gene-PSEN1 were elevated in Cluster 1 (p=6.61e-08, p=7.66e-05, respectively; **Figure 4E**). The beta-secretase coding gene-BACE1, gamma-secretase coding gene-PSEN2 were significantly decreased in Cluster 1 compared to Cluster 2 and healthy agers (p = 1.08e-09, p<2e-16, respectively; **Figure 4E**). Overall, the gene expression of Cluster 2 was intermediate between Cluster 1 and the healthy samples, which may be an intermediate AD disease state between normal and AD. Moreover, concerning the AD Braak stage, there was no difference between the two subclasses (**Figure 4F**). The tissue origin of Cluster 1 and Cluster 2 is shown in **Figure 4H**. The proportions of the entorhinal cortex, middle temporal gyrus, superior frontal gyrus, and post-central gyrus were significantly different in the two subclasses (p=0.0108, **Figure 4G**).

To better understand the biological processes distinguishing the clusters, we performed gene set variation analysis (GSVA). Cluster 1 showed significant enrichment in signal transduction-related pathways, such as olfactory transduction, and neuroactive ligand receptor interaction. In contrast, Cluster 2 was enriched in DNA replication, apoptosis, Fc gamma R-mediated phagocytosis, endocytosis pathways, and *neurotrophin signaling pathway* (**Figure 4H**). Additionally, cell-type identification by estimating relative subsets of RNA transcripts (CIBERSORT) algorithm was applied to investigate the infiltration of 22 types of immune cells in two clusters. Comparing the immune landscape between Cluster 1 and Cluster 2, we found there was a significant difference in 12 immune cell types (**Figure 4I**). Cluster 1 had higher levels of M1 and M2 macrophages, neutrophils, resting NK cells, CD4 memory resting T cells, and regulatory T cells (Tregs). Conversely, Cluster 2 showed increased levels of memory B cells, activated dendritic cells, activated NK cells, plasma cells, CD8+ T cells, and follicular helper T cells. These differences in immune cell infiltration suggest distinct immune landscapes between the two molecular subtypes.

### 3.4 Screening for DEGs in patients in PANoptosis-related molecular subtypes

**Figure 5:**
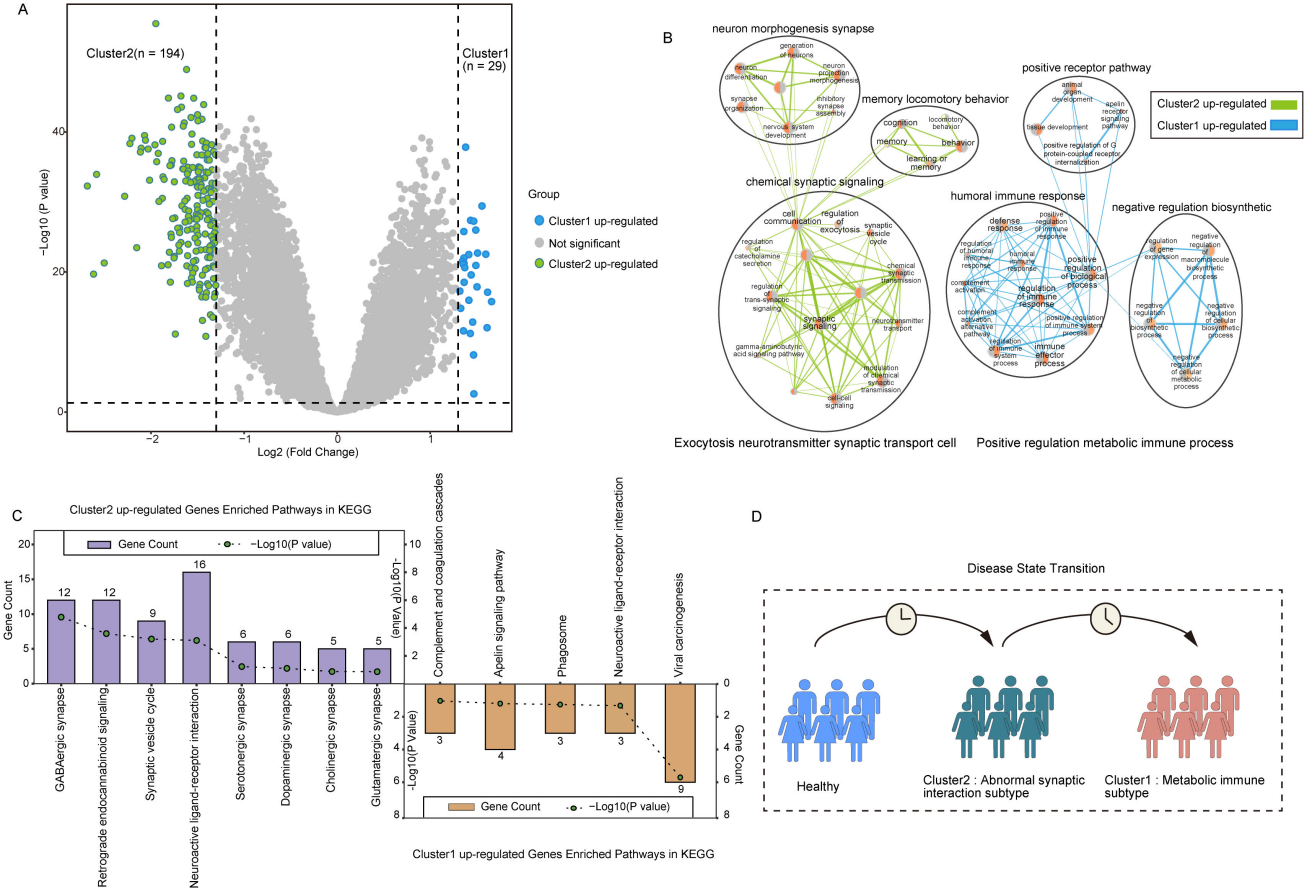
Characterization of PANoptosis-related molecular subtypes. **(A)** The volcano plot for the analysis of differences between the two subtypes. **(B)** Network diagram of biological processes underlying differential gene enrichment in two subtypes. **(C)** Differences in KEGG pathways enriched in the two subtypes. **(D)** Abstract graph of state transfer for two subtypes.

To explore the key genes that differed in the two subpopulations, we screened 223 DEGs in both clusters using the R package ‘Limma’ under the conditions of p-value< 0.05 and | log2(Fold Change) | > 1.3. Of these, 29 genes were up-regulated for expression in Cluster 1, and 194 genes were up-regulated in Cluster 2 (**Figure 5A**).

Analysis of the biological processes of DEGs between the clusters showed Cluster 2 up-regulated genes were mainly enriched in chemical synaptic signaling, neuron morphogenesis synapse, memory locomotory behavior, and other cytosolic neurotransmitter synaptic transporters. Up-regulated genes in Cluster 1 were mainly enriched in positive receptor pathway, humoral immune response, negative regulatory biosynthetic, response defense, and other positive regulatory metabolic immune processes (**Figure 5B**). KEGG pathway enrichment further supported these findings, showing that DEGs in Cluster 2 were significantly involved in pathways such as the GABAergic synapse, neuroactive ligand-receptor interaction, synaptic vesicle cycle, and glutamatergic synapse. Meanwhile, DEGs in Cluster 1 were enriched in immune-inflammatory pathways, including complement and coagulation cascades and the apelin signaling pathway (**Figure 5C**).

Studies have shown that abnormal glutamate-mediated excitatory synaptic transmission and impaired gamma-aminobutyric acid (GABA)-mediated inhibitory synaptic transmission promote early AD seizures and exacerbate cognitive impairment (37). The disruption of the GABAergic system in early AD leads to overactivity of hippocampal neurons, resulting in an imbalance between excitation and inhibition (38, 39). These results indicate that Cluster 2 may represent a transitional state between normal and Cluster 1, signifying the early stage of synaptic dysfunction in AD (**Figure 5D**).

### 3.5 Construction of hub genes within two subtypes and prediction of drug-gene interactions

**Figure 6:**
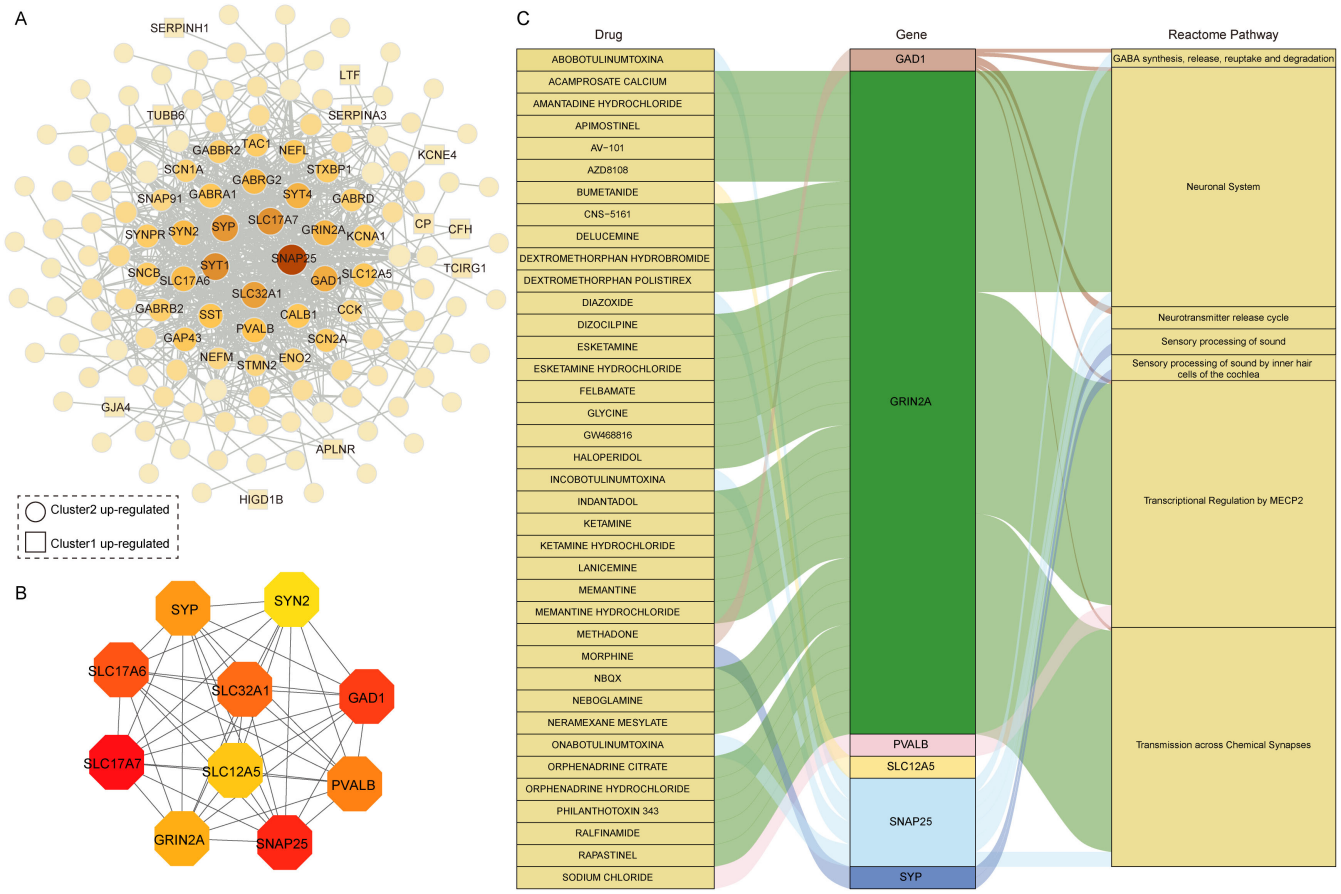
Drug prediction based on hub genes in differentially expressed gene clusters. **(A)** PPI network of 223 DEGs between two distinct clusters. **(B)** The hub genes in the PPI network. **(C)** Drug-gene-pathway interaction prediction of hub genes,6 genes were targeted in the DGIdb database.

To further investigate the interactions of these 223 DEGs between the two AD clusters, we constructed a PPI degree-weighted network with 158 nodes and 706 edges, including 142 Cluster 2 up-regulated genes, and 16 Cluster 1 up-regulated genes (**Figure 6A**). Among them, SNAP25, SLC17A7, SYP, SYT1, and SLC32A1 genes were the top 5 genes with the highest connectivity in the network. Using the MCC algorithm with plug-in CytoHubba (30), we identified the top 10 hub genes with the highest scores: SLC17A7, SNAP25, GAD1, SLC17A6, SLC32A1, PVALB, SYP, GRIN2A, SLC12A5, and SYN2 (**Figure 6B**). All 10 hub genes were upregulated in Cluster 2. Recent studies highlight that SNAP25, a marker of synaptic degeneration, is elevated in the early stages of AD (40), supporting our analysis of Cluster 2 as representing an early AD subtype. Moreover, in the rat model, Grin2a is a synaptic protective factor in astrocytes early in Aβ exposure, and knockdown of Grin2a exacerbates β-amyloid-induced memory and cognitive deficits (41).

Using the DGIdb database, drug-gene interaction analysis revealed 6 hub genes corresponding to 38 potential drug targets and 7 associated pathways for AD treatment (**Figure 6C**, **Supplementary Table S3**). Notably, 30 drugs target GRIN2A, the gene with the highest number of drug predictions, while 4 drugs target SNAP25. GAD1, PVALB, SYP, and SLC12A5 were each targeted by one drug. GRIN2A primarily involves pathways like Transcriptional Regulation by MECP2 and Transmission across Chemical Synapses. MECP2, which interacts directly with RNA polymerase II, plays a crucial role in regulating human neuron transcription (42). Among the predicted drugs, memantine and memantine hydrochloride were classical drugs for Alzheimer’s disease that target GRIN2A. Memantine, a moderate affinity uncompetitive NMDA receptor antagonist that interacts with its target only during states of pathological activation, acts on transmission across chemical synapses pathway (43, 44). Interestingly, many of the predicted drugs are used to treat depression, suggesting a potential link between depression and AD, particularly given the upregulation of these genes in the early AD subtypes. In addition, we analyzed 16 DEGs in Cluster 1 related to drugs, with 6 genes corresponding to 79 drugs and compounds (**Supplementary Figure S4**). Among these, TUBB6 was linked to 41 drugs, CP to 14 drugs, and PRKX to 13 drugs. TUBB6 is a cytoskeletal component involved in metabolic and immune-related processes (45, 46), further supporting its potential as a therapeutic target. These results suggest that two subtypes of hub gene are known drug targets and associated with multiple neuronal system pathways and could be potential candidate targets for AD treatment.

### 3.6 Specificity of cellular distribution of the two subtypes revealed by single cell analysis

**Figure 7:**
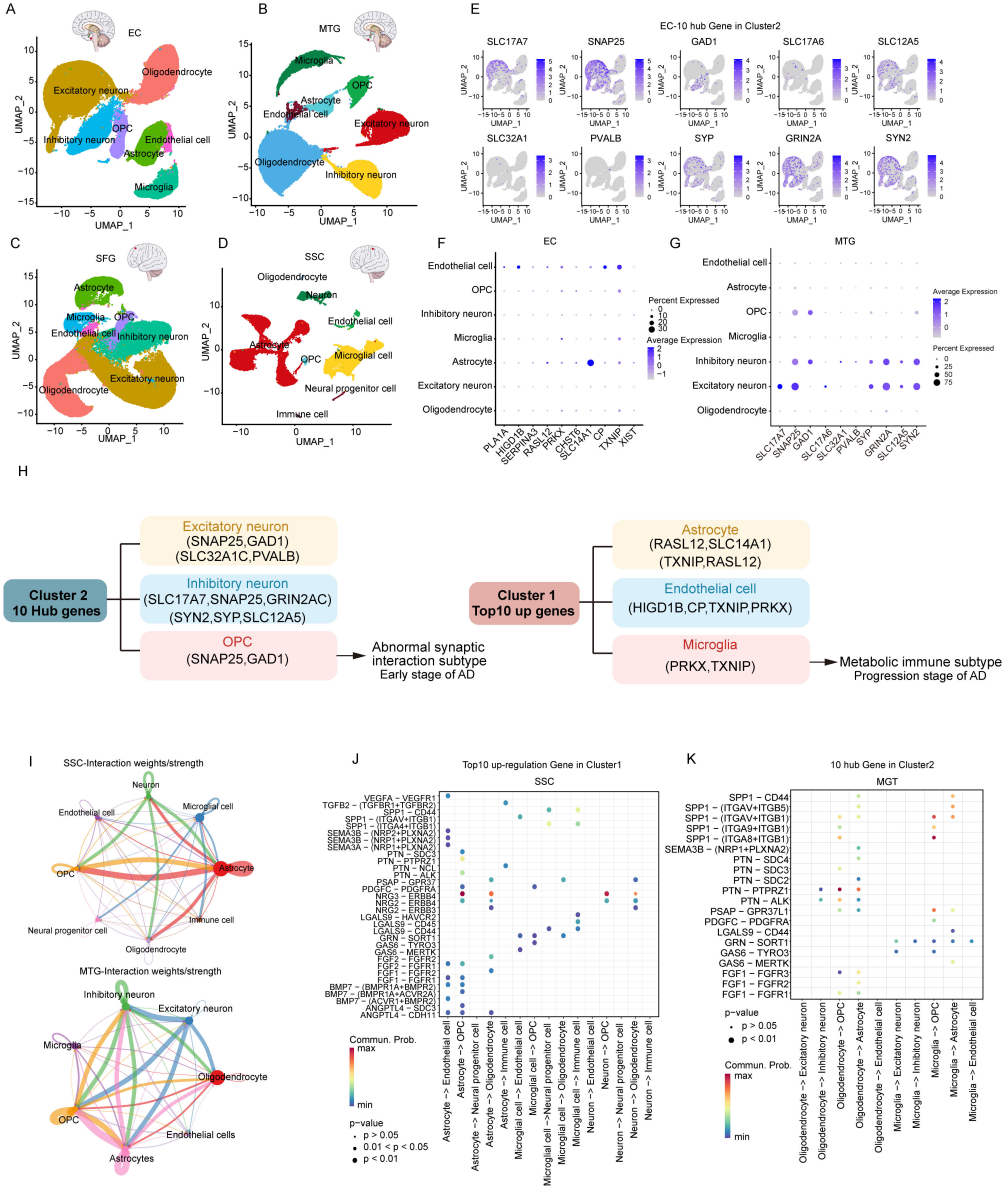
Characterization of key genes of two subtypes based on single-cell data. **(A-D)** UMAP of brain zone across major cell classes including excitatory neuron, inhibitory neuron, oligodendrocyte, oligodendrocyte precursor cell (OPC), astrocyte, immune cell (Immune), microglia, and endothelial cell. **(E)** 10 hub genes of subtype 2 in the EC region. **(F)** top 10 genes of subtype 1 in the EC region. **(G)** 10 hub genes of subtype 2 in the MTG region. **(H)** Distribution of key genes for subtype 1, subtype 2. **(I)** Cellular interactions between major cell classes. **(J)** Bubble heatmap showing cell interaction strength for different ligand-receptor pairs in SSC. Dot size indicates the p-value generated by the permutation test and dot color represents communication probabilities. **(K)** Bubble heatmap for different ligand-receptor pairs in MGT.

To clarify the processes underlying the key genes of the two subtypes during AD, we initially re-analyzed previously published scRNA-seq datasets that included entorhinal cortex (EC), middle temporal gyrus (MTG), superior frontal gyrus (SFG) and somatosensory or entorhinal cortex (SSC) using the Seurat approach. After normalizing gene expression, we performed the PCA for four brain regions and clustered cells using UMAP-based clustering on the informative PCA space, seven distinct cell populations present in EC, MTG, and SFG were identified based on highly variable genes, and eight-cell populations were identified in SSC (**Figures 7A–D**). We identified the seven major classes of cells based on multiple cell markers: excitatory neurons (ExN), inhibitory neurons (InN), microglia (Micr), astrocyte (Astro), endothelial cell (Endo), oligodendrocyte (Oligo), and oligodendrocyte progenitor cell (OPC). In addition, Neural progenitor cell and immune cell marker genes were found in SSC. The proportions of distinct cell types in each region are depicted in **Supplementary Figures S5A–D**, revealing that oligodendrocyte cells, excitatory neuron cells, astrocyte cells, and inhibitory neurons were abundant in EC, SFG, and MTG. However, astrocyte cells and microglial cells were most abundant in SSC.

Next, we analyzed the expression distribution of 10 hub genes within various cells in EC (**Figure 7E**). The results revealed that almost all hub genes were expressed in excitatory neurons and inhibitory neurons, especially SNAP25, the most critical hub gene, which was strongly expressed. GAD1 was specifically overexpressed in inhibitory neurons, whereas SLC32A1 and PVALB were less distributed among seven cell types. However, the results of the expression distribution of the top 10 Cluster 1 upregulated genes showed that most of the genes were expressed in endothelial cells and astrocytes. SLC14A1 was strongly expressed in astrocytes, whereas TXNIP was expressed in endothelial cells, microglia, OPCs, and astrocytes (**Figure 7F**). Consistently, Cluster 2 hub genes showed the same expression distribution in MTG and SFG (**Figure 7G**, **Supplementary Figures S5E, F**). Inconsistently with EC, the up-regulated gene XIST in Cluster1 was expressed in all types of neuronal cells in MTG. In SSC, Cluster 2 hub genes were predominantly expressed in neurons, especially SNAP25, GAD1, GRIN2A, and SYN2. Among the Cluster 1 up-regulated genes, PRKX and TXNIP were highly expressed in immune cells, and microglia (**Supplementary Figure 6A**). In conclusion, our analysis revealed that the Cluster 2 hub genes tend to be expressed in excitatory neurons, inhibitory neurons, and OPC cells, whereas the Cluster 1 top10 genes are widely expressed in astrocytes, endothelial cells, and microglia (**Figure 7H**).

### 3.7 Interactions between various types of neuronal cells are explained by cellular communication

To explore the interactions across various types of neuronal cells, we then used CellChat to infer cell-cell communication among various types of neuronal cells. Circle diagrams showed the general strengths of interactions (proportion) between two cell groups to visualize the integrated cell communication networks (**Figure 7I**, **Supplementary Figures S6B, C**). Different cell groups had obviously different contributive signals on the incoming and outgoing signals. Signaling across neuronal cells was consistently enhanced in the SSC and MTG. (**Figure 7I**). Astrocytes, microglia, and neurons contributed the most to both incoming and outgoing signals within the SSC, while excitatory neurons, inhibitory neurons, astrocytes, and oligodendrocytes exhibited the highest communication frequency in the MTG, as well as in the EC and SFG (**Supplementary Figures S6B, C**).

Next, we investigated cell-cell communication across various neuronal cells by modeling ligand-receptor interactions. Calculating the interaction strength for ligand-receptor pairs in distinct cell types, we inferred cell state-specific ligand-receptor interaction networks of four brain regions (**Figures 7J, K**, **Supplementary Figures S6D, E**). For instance, neurons, including excitatory and inhibitory types, expressed high levels of ligands such as NRG2 and NRG3, while their corresponding receptor, ERBB4, was predominantly expressed in oligodendrocytes and OPCs. This indicates that these ligands may play an important role in influencing signaling between neuronal cells.

**Figure 8:**
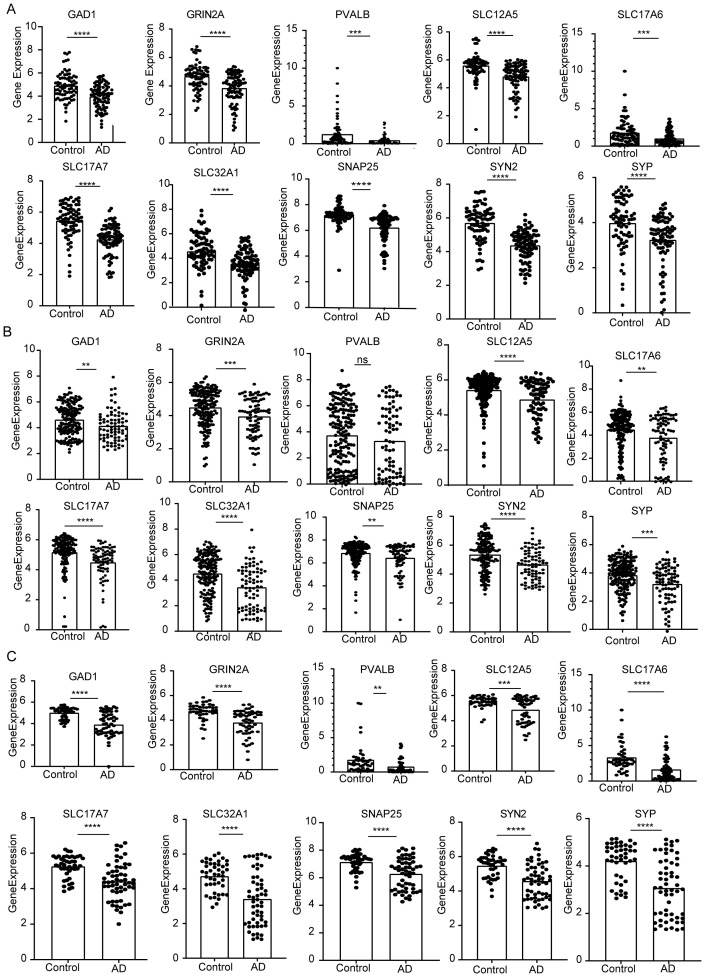
The expression of the identified top 10 hub genes (GAD1, GRIN2A, PVALB, SLC12A5, SLC17A6, SLC17A7, SLC32A1, SNAP25, SYN2, SYP). **(A)** mRNA expression of hub genes in GSE5281. **(B)** GSE48350. **(C)** GSE122063. P values were shown as: **P< 0.01; ***P< 0.001; ****P< 0.0001, ns represent no significant.

At last, 10 potential targets in the Cluster 2 mentioned above were verified by GEO datasets. We detected the expression of those genes in samples of AD patients using GSE48350, GSE122063, and GSE5281 datasets (**Figure 8**). The results showed that these 6 genes were consistently downregulated and expressed in AD. In contrast, PVALB showed no significant change in expression in the GSE48350 dataset. Notably, GRIN2A and SNAP25, which are linked to several predicted drug targets, were significantly downregulated across all three datasets. The downregulation of these genes in the early subtypes of AD suggests that these genes have an inhibitory effect on AD progression.

## 4 Discussion

Cells undergo multiple programmed cell death procedures through a wide range of crosstalk that can be activated simultaneously in the context of specific conditions, such as PANoptosis (“P,” pyroptosis; “A,” apoptosis; “N,” necroptosis); (47, 48). Previous studies of target molecules in the therapeutic PCD pathway have been largely unsuccessful, possibly owing to functional redundancy among them (49, 50). Therefore, targeting PANoptosis and its regulatory complex, the PANoptosome, could offer novel therapeutic strategies for Alzheimer’s disease (AD) (51, 52). Apoptotic and necrotic cells are present in AD and could be simulated in models of AD neuronal degeneration (53, 54). For AD patients, high expression levels of inflammasome (e.g., NLRP1, NLRP3, and AIM2) could activate inflammatory factors, leading to the death of neuronal cells via pyroptosis (55–58). Given the complexity of AD’s pathomechanisms, understanding the interplay between apoptosis, necroptosis, and pyroptosis is critical to advancing AD research (59). Although research on PANoptosis and AD is still limited, the association between apoptosis, pyroptosis, necroptosis, and AD is becoming clearer.

In the study, we found that 24 PANoptosis genes were differentially expressed in AD patients compared with control samples, suggesting that PANoptosis genes may be important indicators of AD progression. Additionally, we found strong correlations between PANoptosis genes and well-established AD susceptibility genes, including PSEN1, PSEN2, APP, and MS4A6A, which are linked to CD44, IL18, and TNFRSF10A. Using logistic regression, all 24 genes were implicated in influencing AD progression, and nine were selected as key variables in our final model. Among these, HGF, YWHAZ, and TNFRSF10B were identified as significant contributors. Notably, TNFRSF10B, a known risk gene for AD, is involved in glycolytic metabolic pathways and inflammatory responses. The pathogenesis of AD involves inflammatory activation and metabolic reprogramming of microglia, especially the metabolic shift from oxidative phosphorylation to glycolysis (60), highlighting the connection between inflammation, metabolic dysregulation, and AD pathogenesis.

The pathological features of AD were the cleavage of AβPP to Aβ to form amyloid plaques and intraneuronal neurogenic fibril tangles (NFT). Three major secretases ADAM10, BACE1 and γ-secretase (including PSEN1, PSEN2), and MAPT are involved in this process (61–63). In this study, we identified two distinct AD states based on the PANoptosis gene. Compared to subtype 1 and the normal subgroup, the expressions of MAPT, ADAM10, BACE1, and PSEN2 in subtype 2 resided in an intermediate state, which implied that subtype 2 might be a transition state from normal to AD. However, the expression of the PSEN1 gene was not intermediate between subtype 1 and normal. Based on the differential genes of the two clusters, we found that Cluster 2 was mainly associated with exocytosis neurotransmitter synaptic transport cell, GABAergic synapse, and neuroactive ligand-receptor interaction, while Cluster 1 is associated with phagosome, complement, and coagulation cascades inflammatory pathways. This finding provides further evidence that subtype 2 is an early AD stage. Qing et al. found that GABAergic abnormalities occur early in AD, earlier than cognitive deficits. Brain regions in AD, such as the hippocampus, exhibit early and tonic hyperexcitability, which may be associated with damage to GABAergic circuits (38). In addition, single cell sequencing data showed that Cluster 2 hub genes were mainly highly expressed in neuronal cells, and Cluster 1 highly expressed genes were more inclined in astrocyte, endothelial cells. The results indicate that there might be abnormalities in the glutamatergic synapse as well as GABAergic synapse in the early stage of AD, leading to the disruption of neurotransmitter transmission among neurons and causing excitation or inhibition of synaptic neurons, and ultimately resulting in brain function abnormalities.

In addition, with Cluster 2 hub genes, we identified six genes with potential target drugs or compounds, among which GRIN2A corresponds to more than 30 drugs. GRIN2A encodes the GluN2A subunit of the NMDA (N-methyl-d-aspartate) receptor (NMDAR), which plays a crucial role in excitatory synaptic transmission and plasticity (64, 65). Most of the drugs corresponding to GRIN2A are antidepressants as well as antipsychotics, such as apimostinel, esketamine, and haloperidol, which improve cognitive deficits caused by the early stages of Alzheimer’s disease (66, 67). These findings suggest that hub genes, such as GRIN2A, play a pivotal role in modulating neurotransmission and may serve as promising therapeutic targets for Alzheimer’s disease.

To the best of our knowledge, this is the first study to characterize early subtypes of AD from a PANoptosis perspective. The screening and validation of these characterized genes provide potential molecular targets for further exploration of the mechanism of programmed cell death in AD. The three GEO datasets analyzed may not fully represent the entire Alzheimer’s disease patient population, thus potentially failing to capture the full spectrum of AD heterogeneity. Furthermore, while we focused primarily on AD marker genes within the context of APANRGs, a deeper investigation into the regulatory mechanisms of each PANoptosis gene associated with AD is necessary. This will be a key focus of our future research efforts.

In conclusion, our comprehensive bioinformatics analysis revealed a strong association between PANoptosis genes and AD pathogenesis. Two subclasses of AD from the perspective of pan-apoptosis were identified with substantial differences in clinical features, metabolic characteristics, and immune infiltration. The results may better elucidate the heterogeneity of AD patients. Additionally, we highlighted 10 hub genes, including SLC17A7, SNAP25, GAD1, and GRIN2A, as potential markers for early AD diagnosis, offering a more accurate framework for identifying early-stage AD patients.

## Data Availability

The original contributions presented in the study are included in the article/**Supplementary Material**. Further inquiries can be directed to the corresponding authors.
