# Supplementary material for: Identification of early Alzheimer’s disease subclass and signature genes based on PANoptosis genes
Source: Front Immunol. 2024 Nov 22;15:1462003. doi: 10.3389/fimmu.2024.1462003 (PMC11621049; doi:10.3389/fimmu.2024.1462003)
Supplement: Supplementary file 1 [file DataSheet1.docx]

Supplementary Material

# Supplementary Figures and Tables

## Supplementary Figures


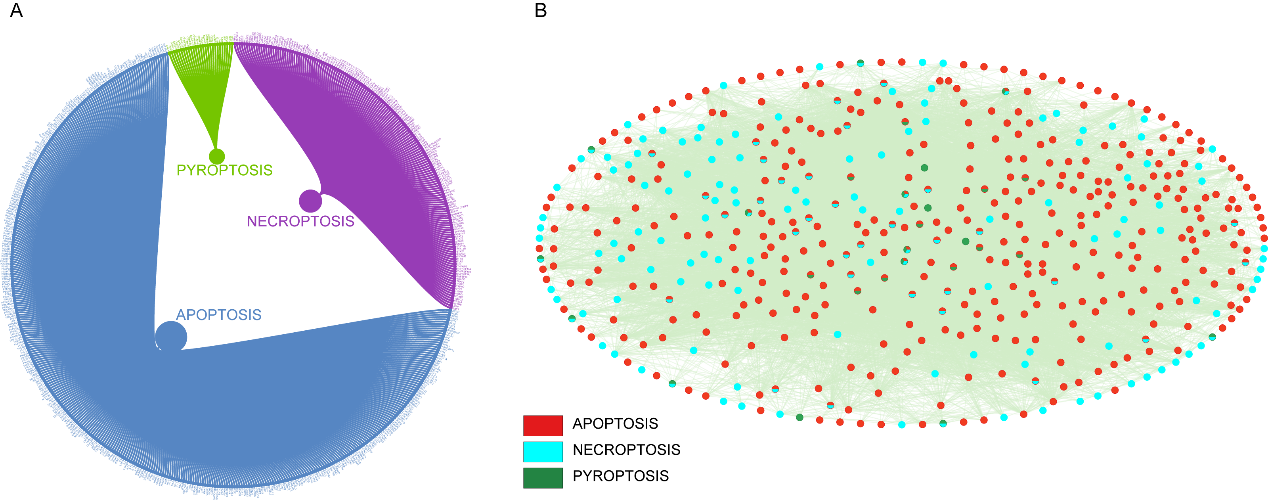


**Supplementary Figure 1**. The characterization of PANoptosis gene. A. PANoptosis genes. B Interaction networks of PANoptosis genes.


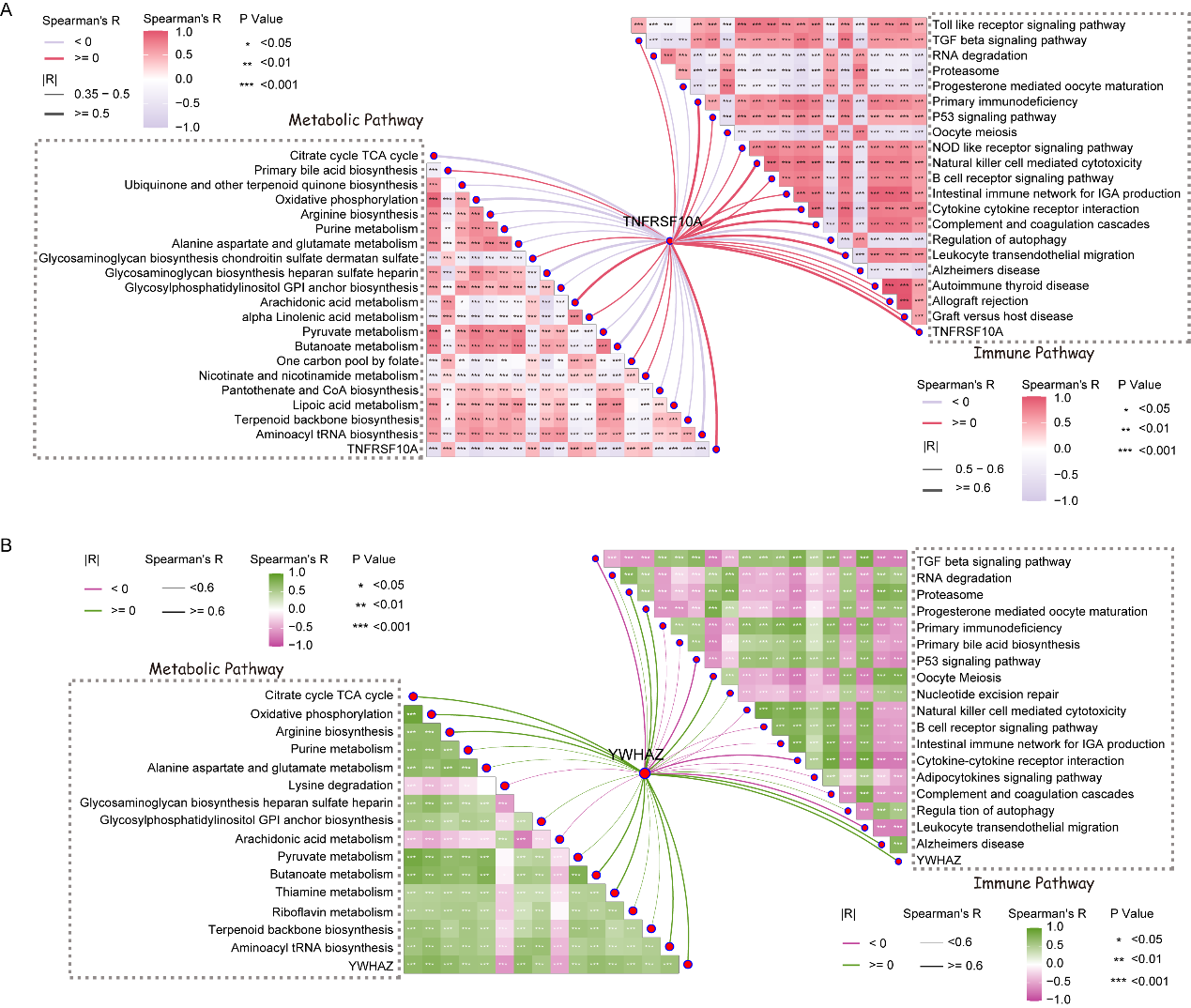
**Supplementary Figure S2.** Enrichment analysis of TNFRSF10A and YWHAZ in metabolic and immune pathways. A. TNFRSF10A gene. B. YWHAZ gene.


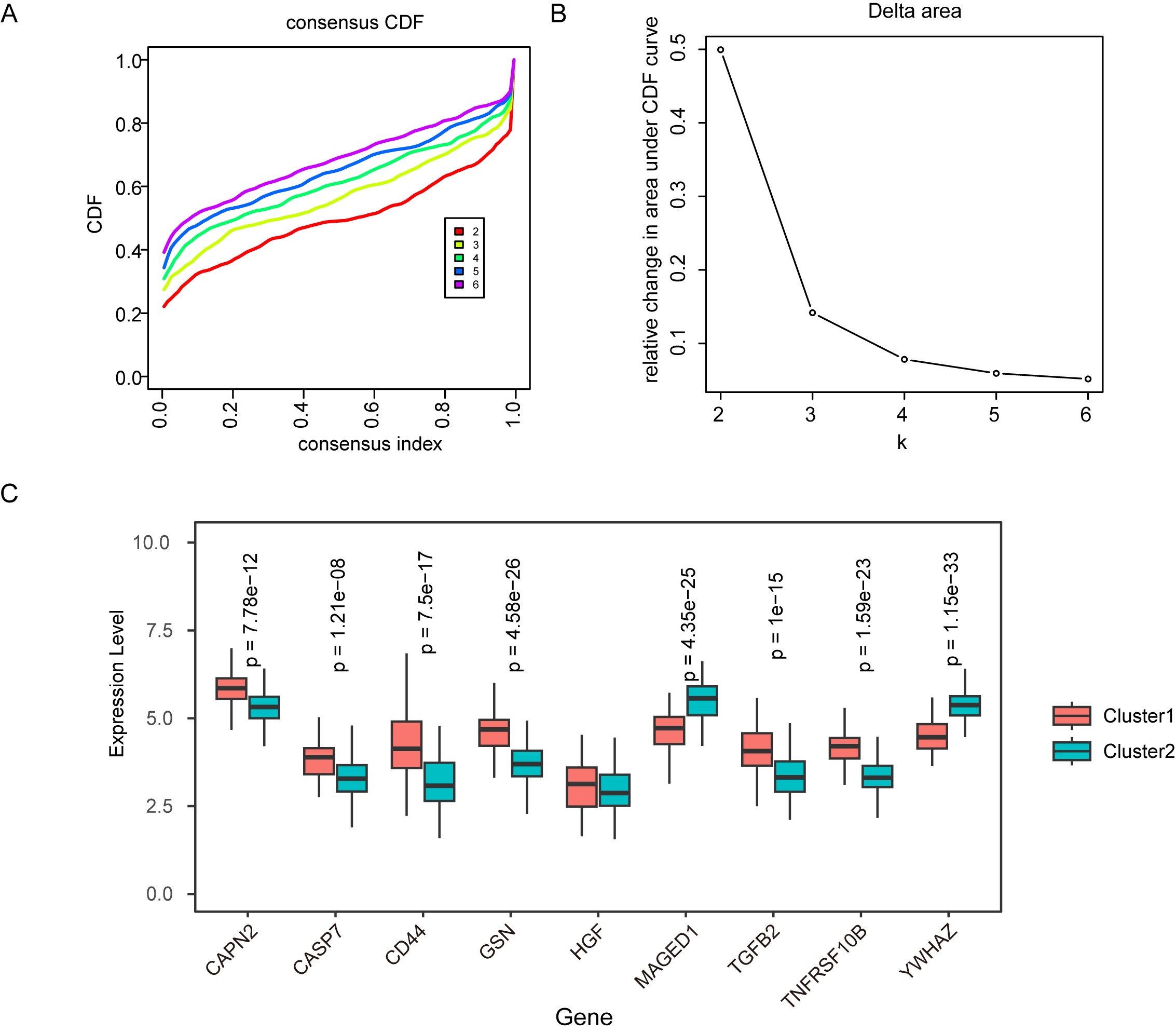


**Supplementary Figure 3.** Identification and analysis of different molecular subtypes of AD based on PANoptosis genes. A. Consensus among clusters for each category number k. B Delta area curves for consensus clustering indicate the relative change in area under the cumulative distribution function (CDF) curve for each category number k compared to k-1. The horizontal axis represents the category number k and the vertical axis represents the relative change in area under CDF curve. C. A box plot showed the expression of the 15 PANoptosis DEGs.


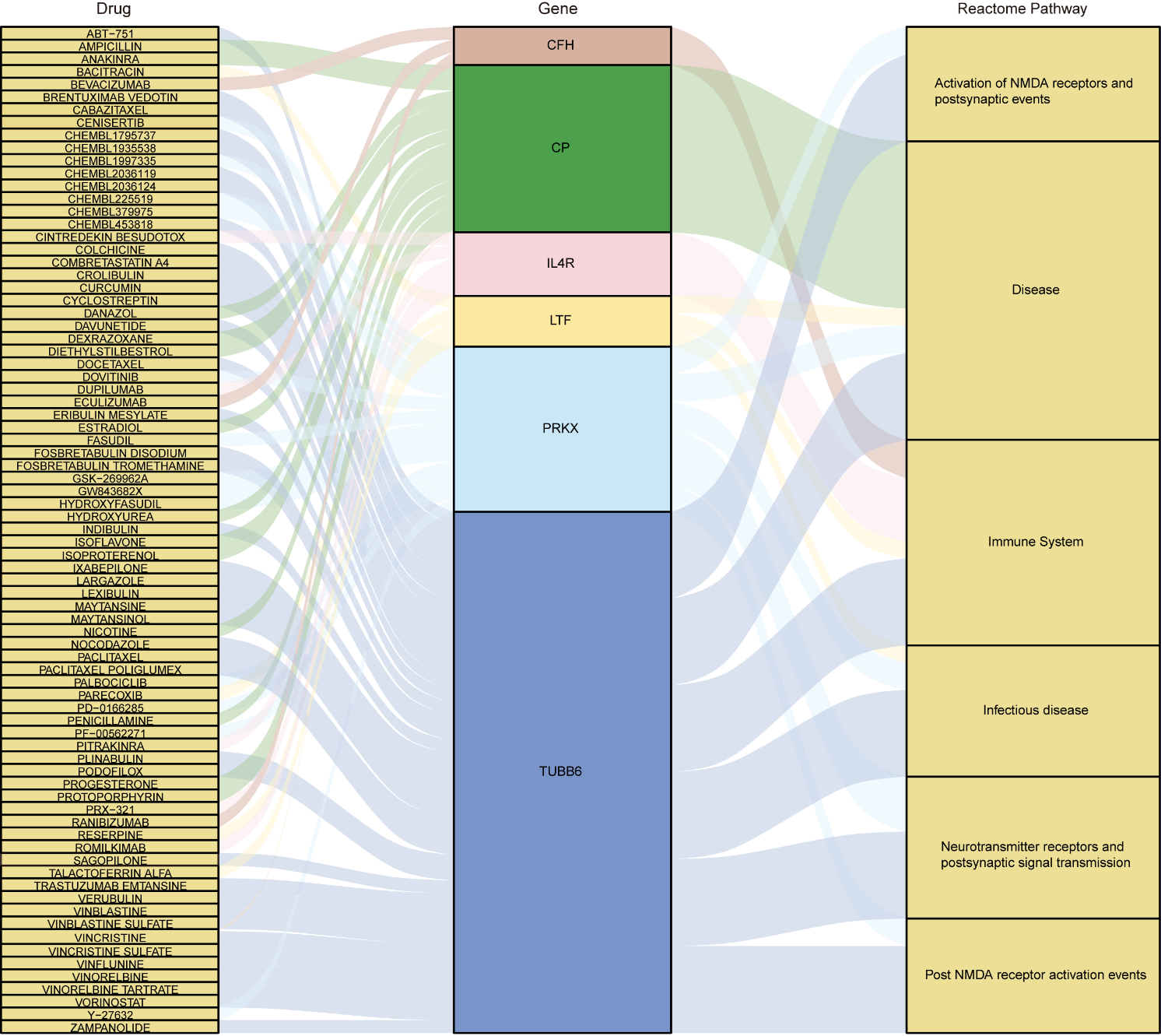


**Supplementary Figure 4.** Drug-gene-pathway interaction prediction of cluster 1 top 10 DEGs. 6 genes were targeted in the DGIdb database.


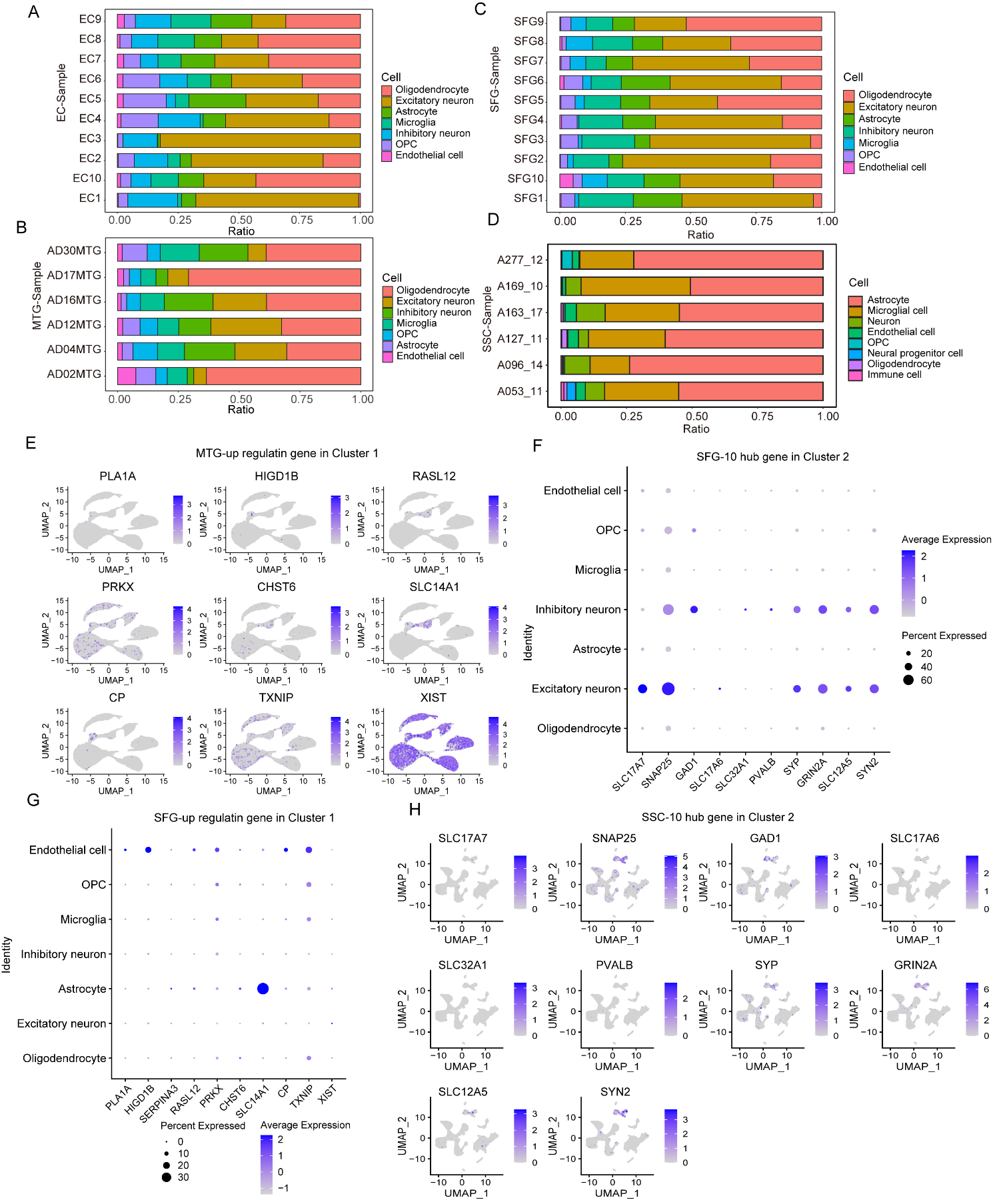


**Supplementary Figure 5.** Characterization of key genes of two subtypes based on single-cell data. A-D. The proportions of distinct cell types in each region,including EC,MTG,SFG and SSC. E. Top10 genes of subtype 1 in the MTG region. F. 10 hub genes of subtype 2 in the SFG region. G. Top10 genes of subtype 1 in the SFG region. H. 10 hub genes of subtype 2 in the SSC region.


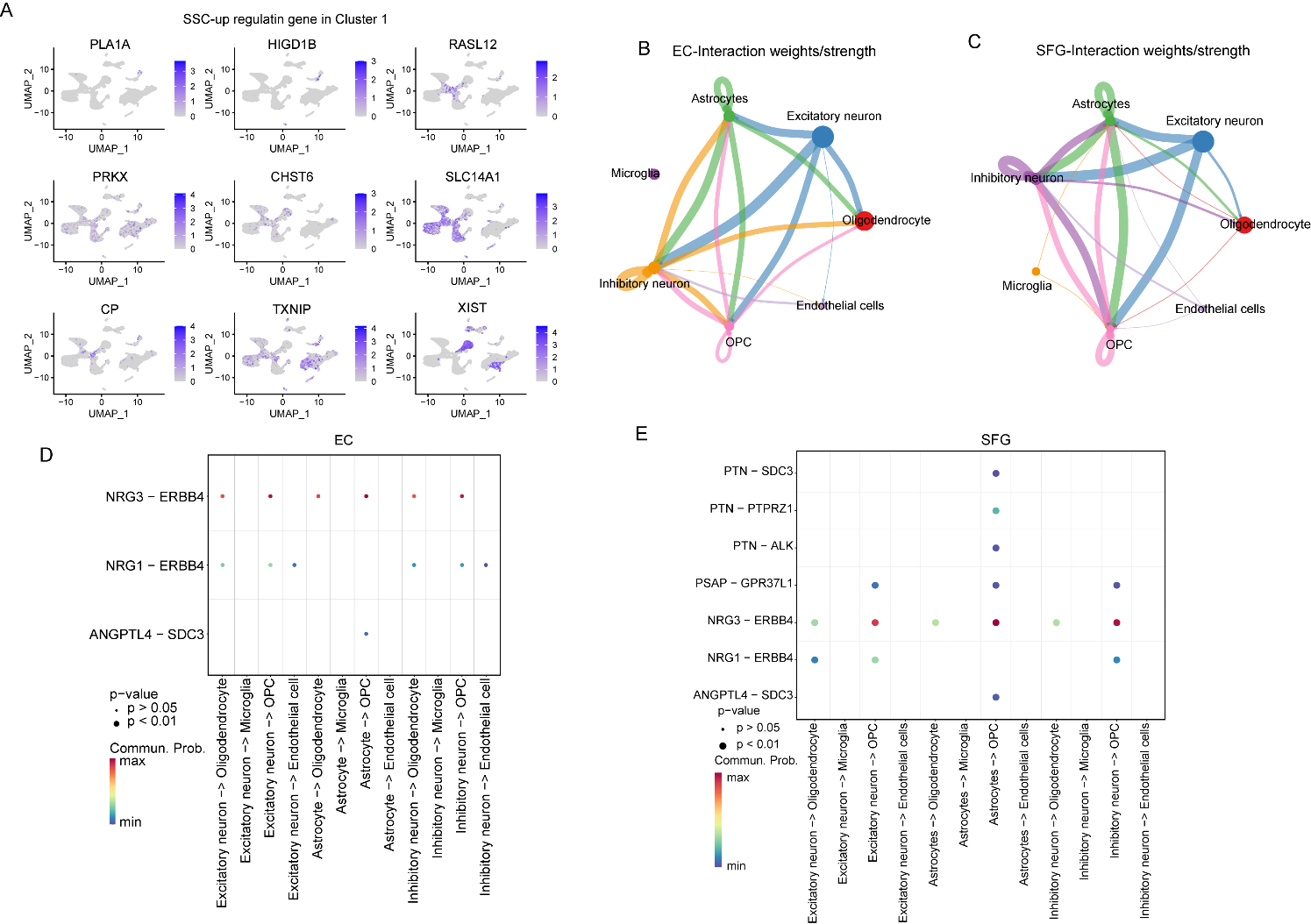


**Supplementary Figure 6.** Analysis of subtype differences in single-cell data. A. Top 10 genes of subtype 1 in the SSC region. B. Cellular interactions between major cell classes in EC region. C. Cellular interactions between major cell classes in SFG region. D. Bubble heatmap showing cell interaction strength for different ligand-receptor pairs in EC. Dot size indicates the p-value generated by the permutation test and dot color represents communication probabilities. K. Bubble heatmap for different ligand-receptor pairs in SFG.

## Supplementary Tables

Table S1 GEO datasets referenced in this study.

Table S2 PANoptosis genes.

Table S3 10 hub gene - drugs interaction.
